# Emotional Speech Recognition Using Deep Neural Networks

**DOI:** 10.3390/s22041414

**Published:** 2022-02-12

**Authors:** Loan Trinh Van, Thuy Dao Thi Le, Thanh Le Xuan, Eric Castelli

**Affiliations:** 1 Faculty of Computer Engineering, School of Information and Communication Technology, Hanoi University of Science and Technology, Hanoi 10000, Vietnam; loan.trinhvan@hust.edu.vn; 2 Department of Software Engineering, Faculty of Information Technology, University of Transport and Communications, Hanoi 10000, Vietnam; thuydtl@utc.edu.vn; 3 LIG, CNRS, Grenoble INP, Inria, Université Grenoble Alpes, 38000 Grenoble, France; eric.castelli@univ-grenoble-alpes.fr; 4 Institute of Engineering, Université Grenoble Alpes, 38000 Grenoble, France

**Keywords:** emotion, speech, recognition, IEMOCAP, CNN, CRNN, GRU, data augmentation

## Abstract

The expression of emotions in human communication plays a very important role in the information that needs to be conveyed to the partner. The forms of expression of human emotions are very rich. It could be body language, facial expressions, eye contact, laughter, and tone of voice. The languages of the world’s peoples are different, but even without understanding a language in communication, people can almost understand part of the message that the other partner wants to convey with emotional expressions as mentioned. Among the forms of human emotional expression, the expression of emotions through voice is perhaps the most studied. This article presents our research on speech emotion recognition using deep neural networks such as CNN, CRNN, and GRU. We used the Interactive Emotional Dyadic Motion Capture (IEMOCAP) corpus for the study with four emotions: anger, happiness, sadness, and neutrality. The feature parameters used for recognition include the Mel spectral coefficients and other parameters related to the spectrum and the intensity of the speech signal. The data augmentation was used by changing the voice and adding white noise. The results show that the GRU model gave the highest average recognition accuracy of 97.47%. This result is superior to existing studies on speech emotion recognition with the IEMOCAP corpus.

## 1. Introduction

The fact that people have emotional expressions is one of the measures showing that human civilization is the highest. It can be said that only humans have very diverse emotional expressions. The expression of emotions can be through body language, eyes, facial expressions, voice, laughter, etc. Just one of them also corresponds to many different emotional forms. In direct or indirect communication, even if there is no corresponding communication image, the human voice both carries the content to be conveyed and at the same time expresses the emotional state of the person towards the communication content. Robots can do many things better than humans, but currently, the expression of emotions of robots, especially through voices, is far behind that of humans. Therefore, the research on speech emotion recognition plays an important role in promoting advances in human–machine interaction. A significant amount of emotional data with different languages has been built and, emotion-recognition studies have been conducted. Among the emotional corpus, IEMOCAP is multimodal emotional dataset in English and has been used as data for research on emotion recognition. For emotion recognition, multimodal recognition can be combined—for example, by combining speech-signal recognition with image recognition (face recognition and body-language recognition) and natural language recognition with noting exclamation words. In the case of such a combination, a better recognition efficiency will be achieved. It can be said that human interaction is marked by affects (attitudes and emotions) [1]. It is known that attitudes are constructed for each language and culture and must be learned by children or second-language students [2]. After [3], all attitudinal expressions are constructed for a certain language and culture, and they can differ between languages. Some attitudes can be expected to have a universal value, but specific attitudes in one language may not be recognized or may be ambiguous in another language [4]. Cross-cultural studies help to better understand this issue [4,5,6]. In contrast, emotions are more universal and therefore less dependent on language and culture [4].

In our study, we limited ourselves to emotion recognition based only on speech signals, and we will present new research results on using deep neural networks for speech emotion recognition with IEMOCAP. The remainder of the article is organized as follows. An overview of relevant studies is presented in Section 2. Section 3 describes the proposed materials and methods. Results and discussions are given in Section 4, and the final section is our conclusion.

## 2. Related Work

The research in [7] has surveyed and evaluated quite a significant number of studies on speech emotion recognition for different corpuses including IEMOCAP [8]. IEMOCAP was a corpus collected by the Speech Analysis and Interpretation Laboratory (SAIL) at the University of Southern California (USC). IEMOCAP launched in 2008, and since then there have been many studies on emotions using this corpus. In general, for convenience of comparison, most studies performed recognition for the same four emotions even though IEMOCAP has data for nine emotions (happiness, anger, sadness, frustration, surprise, fear, excitement, other, and a neutral state). Those four emotions are anger, happiness, sadness, and neutrality. For the happiness emotion, some studies consider excitement as happiness or combine excitement and happiness into a common emotion called happiness. On the other hand, according to [8], happiness and excitement are close in the activation and valence domain. In our study, excitement is considered happiness. To be able to compare the performance of recognition systems using the IEMOCAP corpus, we used four emotions of IEMOCAP as other studies have done. The construction of a system to identify all emotions by IEMOCAP will be reserved for another study.

In Table 14 (at the end of the article for convenience), we listed emotion-recognition studies with the IEMOCAP corpus, and in the limited scope of this article, we only focus on speech emotion-recognition studies using IEMOCAP speech data. The studies were listed mainly in recent years (2019 to 2021), with the remainder being a small number of studies from 2014 to 2018. From Table 14, we gave the models, the feature parameters, and the achieved recognition accuracy for each study. For the models that were used for emotion recognition, the vast majority of IEMOCAP emotion-recognition studies have used neural-network models. The commonality of the studies listed in Table 14 is that there is no data augmentation for IEMOCAP. In [9], the authors used SVM to recognize four emotions from IEMOCAP with an average accuracy of 71.9%. The studies listed in Table 14 from 2015 to now all used neural-network models. Studies using the LSTM model [10,11,12,13,14,15,16] account for a fairly large proportion of the total number of studies. Besides, studies were using CNN in combination with LSTM [17,18,19,20]. CNN, DCNN, and multi-channel CNN models were used in [21,22,23,24]. A combination of CNN and RNN models to get the CRNN model was used in [25]. For the study in [26], the model used was based on attention-based convolutional neural networks (ACNN).

For the feature parameters that have been used for emotion recognition, some studies combine the features of speech and textual data. Those are the studies in [13,15,21,24,27,28]. There are a large number of studies that have used a spectrogram, a Mel-spectrogram, or a combination of a spectrogram and a MFCC as feature parameters [10,14,17,18,22,23,25,29,30,31,32,33,34]. MFCC or MFSC were used in [15,16,19,23,24,26,35,36]. For [11], feature parameters are log-spectra of short-time Fourier transforms. Besides the feature parameters mentioned above, several other features are used in combination such as chromagram, tonnetz, spectral contrast, pitch, spectral centroid, energy, zero-crossing rate, spectral flux, and spectral roll-off [15,16,19,23,35].

Among the studies listed in Table 14, studies [29,31,32] have higher recognition accuracies. In the following, we present more closely these three studies. The recognition accuracies for four emotions (anger, happy, neutral, and sadness) in [29,31,32] were 95.90%, 83.8%, and 81.75%, respectively. The common point of these studies is that they used CNN, and feature parameters were based on a spectrogram. The authors in [29] assumed that individuals may use different means to express emotions and then that Speech Emotion Recognition (SER) should be conditioned on the speaker identity information. So, one of the major contributions of [29] is that the authors have conditioned emotion classification to speaker identity by using a key-query-value attention called Self Speaker Attention (SSA), which allows computing both self and cross-attribute (relation between speaker identity and emotions) attention scores to focus on the emotion-relevant parts of an utterance. For feature parameters, [29] used the 3-D Log-Melspectrogram that consists of a three-channel input. The first channel is the static of the Log-Mel spectrogram from 40 filter banks; the second and third channels are deltas and delta-deltas, respectively, which can be considered as approximations of the first and second derivatives of the first channel. For evaluations, a 10-fold cross-validation technique was performed. There was no data augmentation in [29].

The main contributions of [32] are that the authors proposed an algorithm using a DCNN to extract emotional features for SER and a Correlation-based Feature Selection (CFS) algorithm, which led to improved accuracy for SER. For data, [32] used a supervised resampling filter to oversample the minority class (oversampling increases the number of samples in the minority class). The authors in [32] applied a ten-fold cross-validation technique to their evaluations. The data were randomly split into 10 equal parts for training and testing processes with a splitting ratio of 90:10. Data augmentation was not applied in [32].

For [31], the authors proposed a novel CNN architecture with special strides rather than a pooling scheme to extract the salient high-level features from spectrograms of speech signals for down-sampling the feature maps rather than the pooling layers. The research in [31] performed data pre-processing where the authors removed the noise through a novel adaptive thresholding technique followed by silent portions removal in aural data. The authors performed utterance-based experiments on SER with a five-fold cross-validation technique. The data were split by 80/20; 80% of the data were used for training and 20% for testing the model. There was also no data augmentation in [31].

## 3. Proposed Materials and Methods

In this section, we present the IEMOCAP corpus for experiments, data augmentation, feature parameters, and deep neural network (DNN) models for our research. The last part of the section is a brief description of the performance parameters used to evaluate the research results.

**Figure 1:**
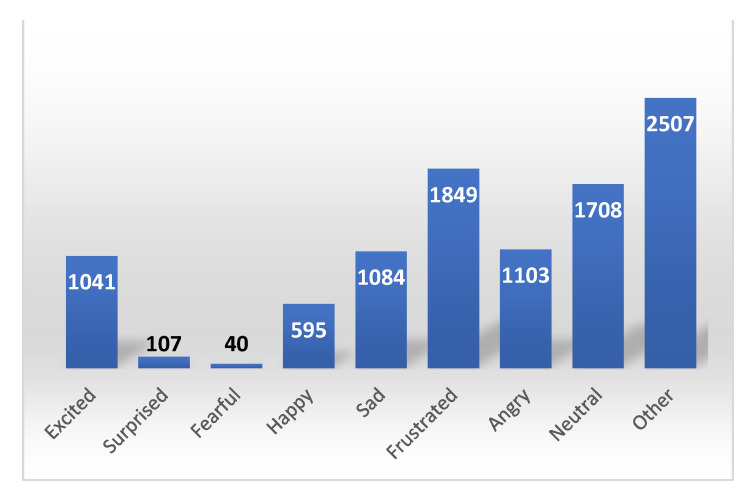
Distribution of sample number for 9 emotions.

IEMOCAP is a multimodal emotional corpus. Ten actors were recorded in dyadic sessions (five sessions with two subjects each). In total, the database contained approximately twelve hours of data. With this database, the authors hoped to be able to expand and generalize their results about the relationship and interplay between speech, facial expressions, head motion, and hand gestures during an expressive speech and conversational interactions. The distribution of the sample number for nine emotions is given in Figure 1.

**Figure 2:**
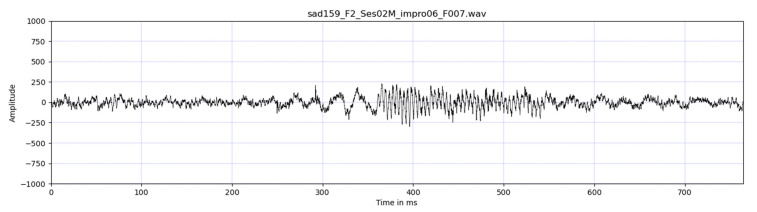
The waveform of a disqualified wav file.

The sampling frequency of IEMOCAP wav files was 16,000 Hz. With a frame width of 256 samples and a frame shift of 128 samples, the average number of frames per wav file was 372 for IEMOCAP wav files. The frame shift was changed according to the sample number of the file. The smaller the number frame in the file, the smaller the frame shift. For the critical case, i.e., where the minimum frame shift was 0, the duration of the corresponding file will then be equal to 256×372/Sampling Frequency=5.952 s. Wav files with a duration less than this value were disqualified. One such case is a wav file whose waveform is shown in Figure 2, the duration of which was 0.7642 s.

After removing corrupted files or files that are too short as mentioned above, the number of wav files of four emotions is as follows: 1075 angry files, 1014 sad files, 1007 happy files, and 1639 neutral files. By using data augmentation as we will show in the next paragraph, the number of files for each emotion were increased by four times. After data augmentation, the sum of files for four emotions was 1075 × 4 + 1014 × 4 + 1007 × 4 + 1639 × 4 = 4735 × 4 = 18,940 files.

In our experiments, the feature parameters were divided into two sets: S1 and S2. The parameter set S1 includes 128 Mel-spectral coefficients. The parameter set S2 includes set S1 plus 25 parameters as shown in Table 1. So, S2 includes 153 parameters. Librosa [37] was used to compute the set of feature parameters S1 and S2.

All 153 parameters can be classified into two groups of parameters: The first group deals with the characteristics of the speech signal spectrum, and the second group is related to the intensity or energy of the speech signal. The first group includes the parameters: Mel-spectral coefficients, spectral flatness, spectral bandwidth, spectral centroid, spectral contrast, roll-off frequency, and pitch. FRMS belongs to the second group. The emotion recognition in this case will be the Mel-spectral image recognition corresponding to the speech signal of the emotion. Therefore, for image recognition, convolution 2D calculation is more appropriate.

The Mel-spectral coefficients are typical characteristics of the different frequencies present in the signal. Spectral flatness is a measure to quantify how much noise-like a sound is. Spectral bandwidth is used to evaluate the spread of the spectrum. For a spectral centroid, each frame of a magnitude spectrogram is normalized and treated as a distribution over frequency bins, from which the mean (centroid) is extracted per frame. To compute the spectral contrast, each frame of a spectrogram is divided into sub-bands. For each sub-band, the energy contrast is estimated by comparing the mean energy in the top quantile (peak energy) to that of the bottom quantile (valley energy). High-contrast values generally correspond to clear, narrow-band signals, while low-contrast values correspond to broad-band noise. Chroma features consist of a twelve-element vector with each dimension representing the intensity associated with a particular semitone, regardless of the octave. The roll-off frequency denotes the approximate low-bass and high-treble limits in a frequency-response curve. Pitch reflects the bass or the treble of the perceived sound, and each sound is emitted with a fixed frequency. The pitch of the sound depends on these frequencies, and the higher the frequency of the sound, the higher the frequency of the perceived sound that will increase and vice versa. FRMS is a root-mean-square (RMS) value calculated for each frame.

Our contribution compared with previous studies on speech emotion recognition to the IEMOCAP corpus is the way the data were augmented. We augmented the data in two ways: changing the voice and adding noise. In this way, the data were increased by four times. Changing the voice is done by formant shifting. Female voices become closer to male voices if their formants are shifted to lower frequencies. In contrast, male voices become closer to female voices if their formants are shifted to higher frequencies. The Praat toolkits [38] were used to move the formants. To change the male voice closer to the female voice, the lift coefficient was 1.1, and to change a female voice closer to a male voice, the reduction factor was 0.909 as recommended by Praat.

**Figure 3:**
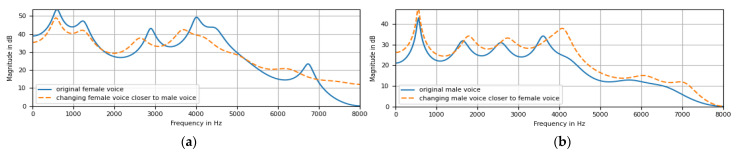
Illustration of the formant translation in two cases: (**a**) consequence of changing female voice closer to a male voice and (**b**) consequence of changing male voice closer to female voice.

Figure 3 illustrates the consequence of changing the voice by shifting the formants. Formants are the harmonic frequencies that occur in the human voice. They define timbre and change the perception of how a voice has been performed by a vocal conduit. They are characteristic of the “tone of voice “or “timbre” of each sound source and can produce interesting effects by altering them, such as making a man’s voice sound like that of a woman, and vice versa. It is well known that among the formants that can be from F1 to F5, the formants F1 and F2 play an important part in conveying the content of the speech. Higher formants are involved in creating the tone of the voice. So, it can be seen that the shifting of formants is mainly for higher-order formants in terms of consequences.

**Figure 4:**
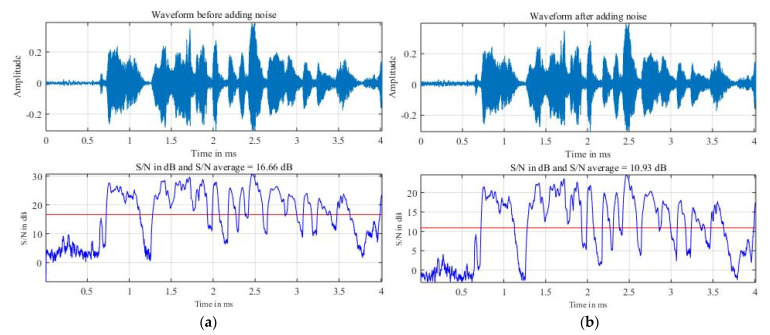
Illustration of waveform and average signal/noise ratio before and after white noise addition. (**a**) Before adding noise. (**b**) After adding noise.

White noise was added to the speech signal using Librosa. The white-noise amplitude was 3% of the maximum amplitude of the speech signal. Figure 4a,b illustrates the waveform and average signal-to-noise ratio before and after noise addition. The red line is S/N average. It can be seen that after adding noise, on average, the S/N ratio decreased by about 5.73 dB. The ratio S/N was calculated according to the following Formula (1):

(1) $${\left(\frac{S}{N}\right)}_{dB}=10log_{10}\left(\frac{P_{S}}{P_{N}}\right)$$

where $P_{S}\;$ is the signal power, and $P_{N}$ is the noise power. Since the signal power is constant before and after noise addition, the noise power ratio before and after noise addition will be:

(2) PN after adding noise PN before adding noise =105.7310≈3.74

This means that on average, the noise power after adding noise increased by about 3.74 times.

In our case, emotion recognition from speech signals became image recognition. Feature parameters were considered as feature images for each emotion. The number of image elements for each image was equal to the product of the number of parameters and the number of frames for a wav file. The number of Mel-spectral coefficients was taken as 128, and the average of the number of frames of the wav files was 372. The frame displacement will change appropriately depending on the size of the wav file so that the total number of frames is constant for all wav files. The reduction of the emotion-recognition problem to the image-recognition problem as mentioned leads to the selection of a model to perform the recognition. Three deep neural-network models, CNN, GRU, and CRNN, were used in our experiments. In the simplest terms, a neural network with some degree of complexity, usually with at least two layers, is qualified as a deep neural network. The emotion recognition in this case can be visualized as similar to image recognition in the following way. Assume that each wav file has n frames of the speech signal. These n frames correspond to n columns of the image. Each frame contains m feature parameters of the speech signal corresponding to that frame. Each feature parameter corresponds to a “pixel” of the image, or each column of the image has m elements on m rows, and each element is a pixel. So, the image to be recognized will have m(rows) × n(columns) pixels.

In a traditional CNN, the input image is passed through the network to extract feature mappings in turn, and finally, we predicted labels on the output in a way where the forward pass is pretty straightforward. Except for the first convolutional layer whose input is the image to be recognized, each layer takes the output of the previous layer to create a feature map in the output, and this feature map is then passed to the next convolutional layer. If the CNN network has L layers, we will have L connections, which are connections between one layer and its next layer. The basic equation for CNN can be expressed as follows:

(3) Y=X⨂W

where X is the input matrix, W is the kernel, Y is the feature map, and ⨂ is convolution. Convolution consists of three operations: addition, multiplication, and shift. We know that the formula for the output signal y(n) for a discrete signal-processing system is as follows, where x(n) is the input signal and h(n) is the impulse response [39]:

(4) $$y\left(n\right)=\overset{\propto }{\underset{k=-\propto }{\sum }}x\left(k\right)h\left(n-k\right)=x\left(n\right)*h\left(n\right)$$

The convolution * in the Formula (4) also includes three operations: addition, multiplication, and shift, and the essence of this convolution is that the signal-processing system has performed the input-signal filtering. Thus, it can be seen that essentially there is a similarity between Formulas (3) and (4). If a CNN is used for image recognition, the convolution in Formula (3) is to filter or extract features of the image. The CNN model used in this study is inspired by [40].

**Figure 5:**
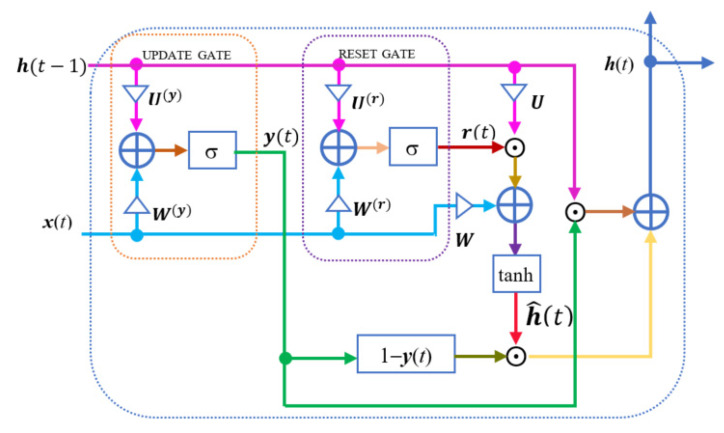
Gated Recurrent Unit.

Recurrent neural networks (RNNs) [41] have a feedback loop from output to input by which the network has memory properties, inferring the next, which is partly based on the previous one. However, the memory capacity of the RNN is inversely proportional to the distance [42]. Long Short-Term Memory (LSTM) is an improvement of RNN, proposed in 1997 by Hochreiter and Schmidhuber [43] to overcome the limitation of the short-term memory capacity of RNN and the vanishing gradient problem. A variant of LSTM is the Gated Recurrent Unit (GRU) [44] because both are designed similarly and, in some cases, produce equally excellent results. The GRU is shown in Figure 5.

The GRU uses and updates the gate and resets the gate to overcome the vanishing gradient problem of a standard RNN. These gates create two vectors that decide what information should be passed to the output. In that way, these gates allow training with long-term information retention without vanishing gradients or discarding information that is no longer suitable for prediction. The update gate helps the model determine the amount of past information (from previous time steps) that needs to be carried forward to the future. This gate executes the following equation at time t:

(5) $$y\left(t\right)=\sigma \left(W^{\left(y\right)}x\left(t\right)+U^{\left(y\right)}h\left(t-1\right)\right)$$

x(t) is the information at time t and is fed into the network and multiplied by the weight $W^{\left(y\right)}$. h(t−1) stores the information of the previous blocks and is then multiplied by the weight $U^{\left(y\right)}$. σ is the sigmoid function to compress the result between 0 and 1.

Basically, the reset gate is used in the model to decide how much past information to forget. The output of this gate is represented by the following equation:

(6) $$r\left(t\right)=\sigma \left(W^{\left(r\right)}x\left(t\right)+U^{\left(r\right)}h\left(t-1\right)\right)$$

The form of Equation (6) is similar to Equation (5) but with different weights. The output of the reset gate r(t) will be used to calculate $\hat{h}\left(t\right)$ as follows (the symbol ⊙ stands for Hadamard product):

(7) $$\hat{h}\left(t\right)=\tanh\left(Wx\left(t\right)+r\left(t\right)⊙Uh\left(t-1\right)\right)$$

W and U are weights. The Hadamard product r(t)⊙Uh(t−1) will determine what to remove from previous time steps. Finally, there is an update gate again. This gate will determine what to collect from the current memory contents $\;\hat{h}\left(t\right)$ and what from the previous steps h(t−1) to continue giving h(t).

(8) $$h\left(t\right)=y\left(t\right)⊙h\left(t-1\right)+\left(1-y\left(t\right)\right)⊙\hat{h}\left(t\right)$$

As such, we can see how the GRU stores and filters information using their update and reset gates. The model does not discard new input each time but keeps the relevant information and passes it down to the next time steps of the network. This eliminates the vanishing gradient issue.

The configuration of the CNN and GRU models for 128 parameters is shown in Table 2 and Table 3. The configurations of the two models remained unchanged for 153 parameters. Naturally, the input size for 153 parameters would be (372,153). For the CRNN model, CNN is followed by the RNN. In our case, the RNN consists of LSTM from 1 to 4, each of them having 128 units (Table 4). For our models, the layers of all models used Kera’s library, where the loss function was “categorical cross-entropy”.

In the following, we present in more detail the layers used in the CNN, CRNN, and GRU. For the parameter set S1, the first layer had the input image with size 372×128 (128 Mel-spectrum coefficients × 372 frames).

For the CNN and CRNN, after taking convolution using a moving 3×3 filter with padding, there were 64 feature maps with a size of 372×128.

For each layer, the goal of batch normalization is to achieve a stable distribution of activation values throughout training and thereby yield a substantial speedup in training [45]. ELU speeds up learning in deep neural networks and leads to higher classification accuracies [46]. Max pooling reduces the number of model parameters, also known as down-sampling or sub-sampling, while also making the detection of features invariant to orientation changes or scale [47]. In the end, dropout is considered a method to prevent neural networks from overfitting [48].

To explain how to calculate the number of parameters given in column Param #, we take the CNN model (Table 2) as an example. The remaining cases can be calculated similarly. For convolutional layers, Param # is the number of trainable parameters. If K is the number of input pictures, M is the number of feature maps, and the number of parameters for a convolution operation is M×(K×(moving filter size)+1). For the layer Conv2D-1, M=64, K=1, and moving filter size =3×3, so Param #=64×(1×3×3+1)=640. For the layer Conv2D-2, M=128, K=64, moving filter size =3×3, and Param #=128×(64×3×3+1)=73856.

The number of parameters for all the MaxPooling2D, Dropout, and Flatten layers was 0 for all. The reason is that these layers do not learn anything. For example, what the MaxPooling2D layer does is to reduce the complexity of the model and to extract local features by finding the maximum values for each 2×2 pool. For fully connected layers, the number of parameters equals the product of the number of neurons *nc* in the current layer and the number of neurons *np* on the previous layer plus one (one is the bias term). For example, for the layer Dense-1, nc=128, np=320, so Param #=128×(320+1 )=41088. For the layer Dense-2, nc=4, np=128, and Param #=4×(128+1 )=516.

For BatchNormalization layers [45], each of them has four parameters, which are γ (gamma weights), β (beta weights),$\;\mu_{\beta }\;$(moving mean), and $\sigma_{B}^{2}$ (moving variance). The first two of them are trainable, but the last two are not. For each BatchNormalization layer, the total number of the parameters =4× size of the input layer. For example, the number of parameters of the BatchNormalization-1 layer =4×372=1488. For the BatchNormalization-2 layer, the number of parameters = 4×64=256.

The number of non-trainable parameters comes from BatchNormalization layers, and in Table 2 the number of non-trainable parameters is (1488+2×256+3×512)/2=3536/2=1768.

The following is a brief description of the quantities such as precision, recall, f1-score, and AUC that are given in Tables 6–12 in Section 4. The precision is the ratio  tp/(tp+fp) where tp is the number of true positives and fp the number of false positives. The precision is intuitively the ability of the classifier not to label as positive a sample that is negative. The recall is the ratio tp/(tp+fn)  where tp is the number of true positives and fn the number of false negatives. The recall is intuitively the ability of the classifier to find all the positive samples. The f1−score can be interpreted as a harmonic mean of the precision and recall, where a f1-score reaches its best value at 1 and its worst score at 0. The relative contribution of precision and recall to the f1-score are equal. The formula for the f1-score is: fi-score =2×(precision×recall)/(precision+recall) [49]. The closer the precision, recall, and f1-score values come to 100%, the better. AUC (Area Under Curve) is the measure of the ability of a classifier to distinguish between classes and is used as a summary of the ROC (Receiver Operator Characteristic) curve. The higher the AUC, the better the performance of the model at distinguishing between the positive and negative classes. Examples of ROC curves are given in Figure 7. A higher *X*-axis value indicates a higher number of false positives than true negatives in a ROC curve, while a higher *Y*-axis value indicates a higher number of true positives than false negatives. The ideal ROC curve hugs the top-left corner, indicating a high true-positive rate and a low false-positive rate. The point corresponding to a perfect classifier would lie on the top-left corner of the ROC graph corresponding to the coordinate (0, 1) in the Cartesian plane. Here, the classifier would correctly classify all the positive and negative class points. ROC curves that fall under the area at the top-left corner indicate good performance levels, whereas ROC curves that fall in the other area at the bottom-right corner indicate poor performance levels [50,51].

## 4. Results and Discussion

The experiments were performed on the computer system with the following hardware configuration: Intel Core i7-8700@3.2GHz × 12 threads processor, 32 GB RAM, 2 TB storage, and NVIDIA GeForce RTX 2080Ti 11GB RAM. The following are the software versions used: Ubuntu 19.10 operating system, Python 3.8, Keras: 2.4.3, Tensorflow 2.2.0, Tensorflow-gpu 2.3.0, Praat 6.1.16, and Librosa 0.7.2.

The data were divided into ten folds, and cross-validation was performed. One fold was extracted from ten folds to dedicate for testing. Out of the remaining nine folds, one was extracted for validation and the remaining eight for training. Extracting one fold from eight folds was done in rotation. In our case, cross-validation was chosen because it generally results in a less-biased or less-optimistic estimate of the model skill than other methods, such as a simple train/test split [50]. With 153 parameters, the average training time for one epoch of GRU, CNN, and CRNN models were 17.8, 30.43, and 44.18 s respectively. Thus, on average in this case, the GRU model’s computational speed was the fastest, and the CRNN’s computational speed was the slowest. The computational speed of the CNN model was faster than the CRNN model but slower than the GRU model. For the GRU model and 153 parameters but without data augmentation, the average training time for one epoch was only 1.57 s. So, increasing the data by four times resulted in an increase in training time for one epoch by about 17.8/1.57 ≈ 11.34 times.

Table 5 is the average recognition accuracy for CNN, CRNN, and GRU models. Table 5 shows that the highest achieved recognition accuracy of 97.47% was for the GRU model with 153 parameters. This recognition accuracy (in bold) was superior to that of the other recognition systems listed in Table 14. For the CNN and GRU models, the average recognition accuracy increased with increasing the number of parameters from 128 (S1 set) to 153 (S2 set), but, for the CRNN model, the average recognition accuracy decreased with increasing the number of parameters from 128 to 153. The GRU model with the property of remembering the past to contribute to future inference but avoiding the vanishing gradient appeared to be more advantageous in this case.

Table 6, Table 7 and Table 8 are the precision, recall, f1-score, and AUC for CNN, CRNN, and GRU models with 153 parameters. Table 9, Table 10 and Table 11 are the precision, recall, f1-score, and AUC for CNN, CRNN, and GRU models with 128 parameters.

Overall, the precision, recall, and f1-score values obtained were very similar to the recognition accuracy as we can see from Table 5, Table 6, Table 7, Table 8, Table 9, Table 10 and Table 11, and the AUC values were also quite close to 1. Table 6, Table 7, Table 8, Table 9, Table 10 and Table 11 show that the common point of CNN, CRNN, and GRU models were that the highest precision, recall, and f1-score were achieved with the “sadness” emotion, and the lowest recall and f1-score were for the “happiness” (“excitement”) emotion and for both sets of parameters. The lowest precision was for the emotions of “excitement” or “anger.” These highest and lowest values are bold styled in Table 6, Table 7, Table 8, Table 9, Table 10 and Table 11.

Table 12 is the recapitulation of the average values of accuracy, precision, recall, f1-score, and AUC with the highest and lowest values for the three models using 128 and 153 parameters. As we can see from Table 12, with the parameter set S1, the CRNN model (in bold) always dominated for the highest values, and the GRU model (in italics) dominated the lowest values. These two models were almost interchangeable with the parameter set S2. The GRU model (in bold) always dominated for the highest values, and the CRNN model (in italics) almost dominated the lowest values.

**Figure 6:**
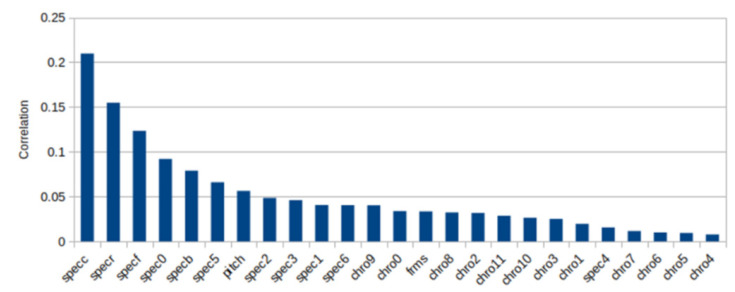
Correlation between 25 parameters and accuracy recognition. specc: spectral centroid, specr: spectral rolloff, specf: spectral flatness, specb: spectral bandwidth, spec0-6: spectral contrast, chro0-11: chroma, frms: root-mean-square (RMS) value calculated for each frame.

From Figure 6 it can be seen that the spectral centroid (“specc” in the figure labels) had the best influence on high recognition accuracy followed by spectral roll-off and spectral flatness.

**Figure 7:**
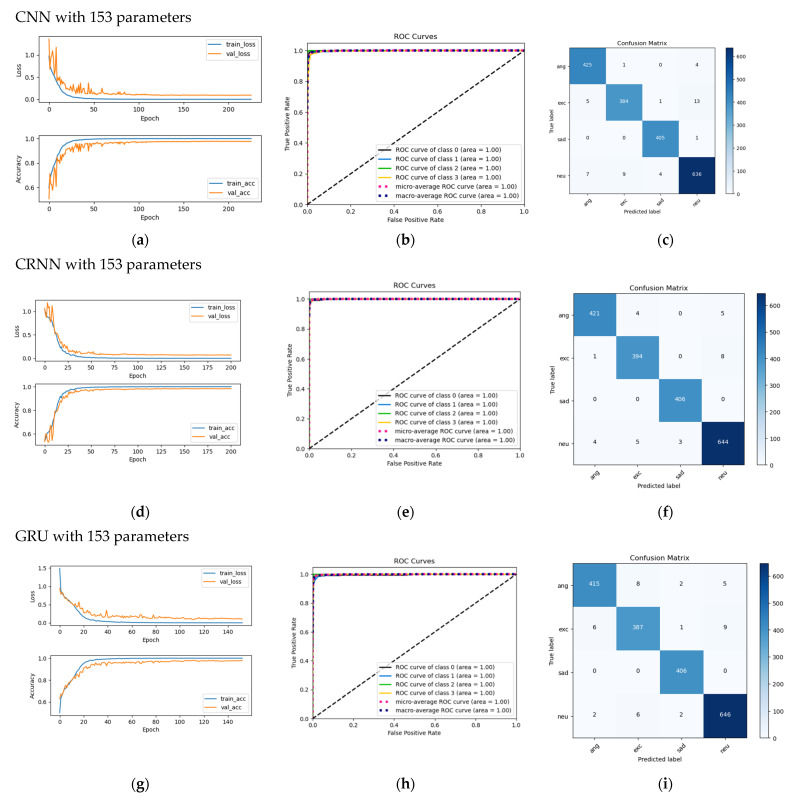
(**a**,**d**,**g**) Examples of loss and accuracy variations according to the epoch for training and validation for a fold; (**b**,**e**,**h**) ROC (class 0: anger, class 1: exc, class 2: sad, class 3: neu.); and (**c**,**f**,**i**) confusion matrix.

Some examples of the variations in loss and accuracy according to the epoch for training and validation and confusion matrices for a fold are shown in Figure 7. Figure 7 shows that variations in validation loss match and variations in training loss. The same was true for validation accuracy and training accuracy. This means there was no overfitting [52].

The confusion matrix in Figure 7 is an example of a test fold. The sum of the elements per row is the number of test samples for each emotion. In this case, the total numbers of samples for the four emotions of anger, excitement, sadness, neutrality were 430, 403, 406, and 656, respectively. The number of samples on the main diagonal corresponds to the true positive (tp) case, which means that each emotion was correctly identified as that emotion. As an example (Figure 7i), for the anger emotion, the sample number for the tp of this emotion was 415. For the false-negative (fn) case, eight anger samples were incorrectly identified as excitement; two anger samples were incorrectly identified as sadness; and five anger samples were incorrectly identified as neutrality. For the false-positive (fp) case, six excitement samples, zero sadness samples, and two neutrality samples were incorrectly identified as anger. For the true-negative (tn) case, (387 + 1 + 9) samples of excitement, 408 samples of sadness, and (646 + 2 + 6) samples of neutrality were correctly identified as not-anger. The cases of fn, fp, and tn were also determined in a similar way for the remaining three emotions.

For comparison, we performed IEMOCAP’s four-emotion recognition with the same GRU model and the same S2 parameter set but without data augmentation. We only chose the GRU model for this experiment because it is the model that gives the best recognition results with data augmentation. In this case, the total number of files for each emotion was reduced by four times (anger: 1075 files, sadness: 1014 files, happiness: 1007 files, and neutrality: 1639 files, as mentioned above). The data division for training, validation, and testing was similar to the case with data augmentation. The results from Table 13 show that on average the accuracy dropped to only 75.83%.

AUC and precision, recall, f1-score for each emotion with the GRU model using 153 parameters without data augmentation, are also given in Table 13. Again, the highest values of precision, recall, and f1-score (in bold) were for the “sadness” emotion, and the lowest values precision, recall, f1-score (in bold) were for the “excitement” or “anger”emotion. However, these values were lower than in the case of data augmentation. The same was true for the mean of AUC.

## 5. Conclusions

In this article, we presented the results of speech emotion recognition with the IEMOCAP corpus. Three deep neural-network models CNN, CRNN, and GRU were used for emotion recognition in our case, and, in general, the GRU model had a slight advantage over the CNN and CRNN models. Data augmentation including changing the voice also contributed to recognition performance. Besides the Mel-spectral coefficients, other spectral features of the speech signal also increased the average recognition accuracy. The results of our research demonstrate that the proposed method outperforms the state-of-the-art methods (see Table 14). For machine learning including deep learning, the more data available, the better the performance of the recognition system. If noise addition is a possible solution for different signal types, then data augmentation by changing the voice is a very specific approach to speech data. Besides choosing the appropriate models and feature parameters, data augmentation also shows its effectiveness especially in cases where the available data are not large enough. Data augmentation increases the amount of memory and the training time, but in return, the performance of the recognition system increases. For the upcoming study, we will be researching emotion recognition on data with a greater number of emotions and combined with emotional speech synthesis. Emotional speech synthesis is also the direction of research where we have achieved some initial results.

## Figures and Tables

**Table 1:** 25 parameters belonging to set S2.

Parameters	Param #
Spectral Flatness	1
Spectral Bandwidth	1
Spectral Centroid	1
Spectral Contrast	7
Chroma	12
Pitch	1
Spectral RollOff	1
FRMS	1

**Table 2:** Configuration of the CNN model with five convolutional layers for 128 parameters.

Model: “sequential”		
Layer (Type)	Output Shape	Param #
BatchNormalization-1	(None, 372, 128, 1)	1488
Conv2D-1	(None, 372, 128, 64)	640
BatchNormalization-2	(None, 372, 128, 64)	256
ELU-1	(None, 372, 128, 64)	0
MaxPooling2D-1	(None, 186, 64, 64)	0
Dropout-1	(None, 186, 64, 64)	0
Conv2D-2	(None, 186, 64, 128)	73856
BatchNormalization-3	(None, 186, 64, 128)	512
ELU-2	(None, 186, 64, 128)	0
MaxPooling2D-2	(None, 93, 32, 128)	0
Dropout-2	(None, 93, 32, 128)	0
Conv2D-3	(None, 93, 32, 128)	147584
BatchNormalization-4	(None, 93, 32, 128)	512
ELU-3	(None, 93, 32, 128)	0
MaxPooling2D-3	(None, 46, 16, 128)	0
Dropout-3	(None, 46, 16, 128)	0
Conv2D-4	(None, 46, 16, 128)	147584
BatchNormalization-5	(None, 46, 16, 128)	512
ELU-4	(None, 46, 16, 128)	0
MaxPooling2D-4	(None, 15, 5, 128)	0
Dropout-4	(None, 15, 5, 128)	0
Conv2D-5	(None, 15, 5, 64)	73792
BatchNormalization-6	(None, 15, 5, 64)	256
ELU-5	(None, 15, 5, 64)	0
MaxPooling2D-5	(None, 5, 1, 64)	0
Dropout-5	(None, 5, 1, 64)	0
Flatten	(None, 320)	0
Dense-1	(None, 128)	41088
ELU-6	(None, 128)	0
Dropout-6	(None, 128)	0
Dense-2	(None, 4)	516
Total params: 488596 Trainable params: 486828 Non-trainable params: 1768		

**Table 3:** Configuration of the GRU model with 128 parameters.

Model: “sequential”		
Layer (Type)	Output Shape	Param #
BatchNormalization	(None, 372, 128)	1488
GRU-1	(None, 372, 256)	296448
Dropout-1	(None, 372, 256)	0
GRU-2	(None, 512)	1182720
Dropout-2	(None, 512)	0
Dense-1	(None, 128)	65664
Activation	(None, 128)	0
Dropout-3	(None, 128)	0
Dense-2	(None, 4)	516
Total params: 1546836 Trainable params: 1546092 Non-trainable params: 744		

**Table 4:** Configuration of the CRNN model for 128 parameters.

Layer (Type)	Output Shape	Param #	Connected to
Input Layer	(None, 128, 372, 1)	0	
Conv2D-1	(None, 128, 372, 64)	640	Input Layer
Conv2D-2	(None, 128, 372, 128)	73856	Conv2D-1
Conv2D-3	(None, 128, 372, 256)	295168	Conv2D-2
BatchNormalization-3 (BN3)	(None, 128, 372, 256)	1024	Conv2D-3
MaxPooling2D-3 (MP3)	(None, 64, 186, 256)	0	BN3
Conv2D-4	(None, 64, 186, 256)	590080	MP3
Conv2D-5	(None, 64, 186, 512)	1180160	Conv2D-4
BatchNormalization-5(BN5)	(None, 64, 186, 512)	2048	Conv2D-5
MaxPooling2D-5 (MP5)	(None, 32, 93, 512)	0	BN5
Conv2D-6	(None, 32, 93, 512)	2359808	MP5
Conv2D-7	(None, 32, 93, 512)	2359808	Conv2D-6
BatchNormalization-7(BN7)	(None, 32, 93, 512)	2048	Conv2D-7
Reshape	(None, 32, 47616)	0	BN7
Dense (Fc9)	(None, 32, 128)	6094976	Reshape
LSTM1	(None, 32, 128)	131584	Fc9
LSTM2	(None, 32, 128)	131584	Fc9
Add	(None, 32, 128)	0	LSTM1
			LSTM2
LSTM3	(None, 32, 128)	131584	Add
LSTM4	(None, 32, 128)	131584	Add
Concatenate	(None, 32, 256)	0	LSTM3
			LSTM4
Dropout1	(None, 32, 256)	0	Concatenate
Flatten	(None, 8192)	0	Dropout1
Dense1	(None, 512)	4194816	Flatten
Dropout2	(None, 512)	0	Dense1
Dense2	(None, 4)	2052	Dropout2
Total params: 17682820 Trainable params: 17680260 Non-trainable params: 2560			

**Table 5:** Average recognition accuracy for CNN, CRNN, and GRU models.

Folds	CNN		CRNN		GRU	
	S1	S2	S1	S2	S1	S2
**0**	96.78	96.83	96.94	94.72	95.04	97.57
**1**	96.57	97.04	96.46	97.20	94.30	96.83
**2**	96.73	97.63	96.09	96.31	96.20	97.84
**3**	96.25	96.83	98.10	95.46	95.25	96.94
**4**	96.57	97.47	95.46	98.31	95.73	97.57
**5**	95.62	96.99	97.68	95.83	95.36	97.52
6	95.73	96.52	98.05	96.41	96.41	97.94
**7**	96.57	96.09	98.94	98.42	95.46	97.73
**8**	96.15	97.20	96.89	97.94	96.04	97.31
**Accuracy Average (%)**	96.33	**96.96**	**97.18**	96.73	95.53	**97.47**

**Table 6:** Precision, recall, f1-score, and AUC for CNN model with 153 parameters.

Folds	Precision				Recall				f1-score				AUC
	ANG	EXC	SAD	NEU	ANG	EXC	SAD	NEU	ANG	EXC	SAD	NEU	
**0**	96.09	97.39	98.54	95.95	97.21	92.56	99.75	97.41	96.65	94.91	99.14	96.67	0.997
**1**	96.53	97.91	99.26	95.54	96.98	92.80	99.75	98.02	96.75	95.29	99.51	96.76	0.997
**2**	97.25	97.46	98.78	97.25	98.84	95.29	99.75	96.95	98.04	96.36	99.26	97.10	0.998
**3**	96.98	97.12	99.27	95.10	97.21	92.06	100.0	97.56	97.10	94.52	99.63	96.31	0.997
**4**	97.24	98.69	99.02	95.98	98.14	93.30	99.75	98.17	97.69	95.92	99.39	97.06	0.997
**5**	95.50	98.40	98.31	96.39	98.60	91.32	100.0	97.56	97.03	94.72	99.15	96.97	0.997
**6**	96.74	95.44	98.54	95.76	96.51	93.55	99.51	96.49	96.62	94.49	99.02	96.13	0.997
**7**	96.29	96.78	99.02	93.84	96.51	89.58	99.75	97.56	96.40	93.04	99.39	95.67	0.996
**8**	97.24	97.15	98.31	96.52	98.37	93.05	100.0	97.26	97.80	95.06	99.15	96.89	0.995
**Average (%)**	**96.65**	97.37	**98.78**	95.81	97.60	**92.61**	**99.81**	97.44	97.12	**94.92**	**99.29**	96.62	0.997
	**97.15**				96.87				96.99				

**Table 7:** Precision, recall, f1-score, and AUC for CRNN model with 153 parameters.

Folds	Precision				Recall				f1-score				AUC
	ANG	EXC	SAD	NEU	ANG	EXC	SAD	NEU	ANG	EXC	SAD	NEU	
**0**	96.19	93.83	95.51	93.82	93.95	90.57	99.51	94.82	95.06	92.17	97.47	94.31	0.995
**1**	96.51	96.98	99.02	96.67	96.51	95.78	99.26	97.26	96.51	96.38	99.14	96.96	0.999
**2**	97.38	97.14	97.34	94.53	95.12	92.80	99.26	97.41	96.24	94.92	98.29	95.95	0.996
**3**	95.33	97.05	98.05	93.12	94.88	89.83	99.26	96.95	95.10	93.30	98.65	95.00	0.996
**4**	99.53	97.75	98.30	97.88	98.14	97.02	99.75	98.32	98.83	97.38	99.02	98.10	0.999
**5**	96.44	96.58	98.30	93.55	94.42	91.07	99.75	97.26	95.42	93.74	99.02	95.37	0.994
**6**	96.69	95.90	98.78	95.10	95.12	92.80	99.51	97.56	95.90	94.33	99.14	96.31	0.997
**7**	98.83	97.77	99.27	98.02	97.91	97.77	100.0	98.17	98.36	97.77	99.63	98.10	0.999
**8**	97.48	97.95	99.02	97.58	98.84	94.79	99.51	98.32	98.15	96.34	99.26	97.95	0.998
**Average (%)**	97.15	**96.77**	**98.18**	**95.59**	96.1	**93.6**	**99.53**	97.34	96.62	**95.15**	**98.85**	96.45	0.997
	**96.92**				96.64				96.77				

**Table 8:** Precision, recall, f1-score, and AUC for GRU model with 153 parameters.

Folds	Precision				Recall				f1-score				AUC
	ANG	EXC	SAD	NEU	ANG	EXC	SAD	NEU	ANG	EXC	SAD	NEU	
**0**	97.42	97.69	100.0	96.16	96.51	94.54	99.01	99.24	96.96	96.09	99.50	97.67	0.995
**1**	98.10	94.58	98.29	96.51	95.81	95.29	99.26	96.95	96.94	94.93	98.77	96.73	0.999
**2**	98.11	96.51	98.78	97.88	96.51	96.03	100.0	98.48	97.30	96.27	99.39	98.18	0.996
**3**	96.90	94.53	99.02	97.15	94.65	94.29	99.26	98.63	95.76	94.41	99.14	97.88	0.996
**4**	99.27	94.66	99.51	97.14	95.35	96.77	99.51	98.32	97.27	95.71	99.51	97.73	0.999
**5**	96.96	96.24	99.75	97.29	96.51	95.29	99.51	98.32	96.74	95.76	99.63	97.80	0.994
**6**	98.35	95.89	99.50	98.02	96.74	98.51	98.77	97.87	97.54	97.18	99.13	97.94	0.997
**7**	98.35	95.58	99.51	97.57	96.74	96.53	99.75	97.87	97.54	96.05	99.63	97.72	0.999
**8**	98.81	96.46	99.26	95.69	96.51	94.54	99.51	98.17	97.65	95.49	99.38	96.91	0.998
**Average (%)**	98.03	**95.79**	**99.29**	97.05	96.15	**95.75**	**99.4**	98.21	97.08	**95.77**	**99.34**	97.62	0.997
	**97.54**				97.38				97.45				

**Table 9:** Precision, recall, f1-score, and AUC for CNN model with 128 parameters.

Folds	Precision				Recall				f1-score				AUC
	ANG	EXC	SAD	NEU	ANG	EXC	SAD	NEU	ANG	EXC	SAD	NEU	
**0**	96.15	96.16	97.83	96.91	98.6	93.3	100.0	95.73	97.36	94.71	98.90	96.32	0.998
**1**	95.66	98.40	97.13	95.78	97.44	91.56	100.0	96.95	96.54	94.86	98.54	96.36	0.997
**2**	96.36	95.65	98.78	96.33	98.37	92.80	100.0	96.04	97.35	94.21	99.39	96.18	0.997
**3**	96.99	95.40	97.36	95.58	97.44	92.56	99.75	95.58	97.22	93.95	98.54	95.58	0.997
**4**	96.53	95.91	98.30	95.91	96.98	93.05	99.75	96.49	96.75	94.46	99.02	96.20	0.997
**5**	93.74	96.77	97.36	95.15	97.44	89.08	100.0	95.73	95.55	92.76	98.66	95.44	0.996
**6**	97.38	96.51	97.83	93.01	95.12	89.33	100.0	97.41	96.24	92.78	98.90	95.16	0.995
**7**	97.18	96.39	98.78	94.94	96.28	92.8	99.51	97.26	96.73	94.56	99.14	96.08	0.998
**8**	96.96	97.60	98.06	93.69	96.28	90.82	99.51	97.26	96.62	94.09	98.78	95.44	0.995
**Average (%)**	**96.33**	96.53	**97.94**	95.26	97.11	**91.70**	**99.84**	96.49	96.71	**94.04**	**98.87**	95.86	0.997
	**96.52**				96.29				96.37				

**Table 10:** Precision, recall, f1-score, and AUC for CRNN model with 128 parameters.

Folds	Precision				Recall				f1-score				AUC
	ANG	EXC	SAD	NEU	ANG	EXC	SAD	NEU	ANG	EXC	SAD	NEU	
**0**	97.18	97.69	97.82	95.81	96.28	94.29	99.26	97.56	96.73	95.96	98.53	96.68	0.998
**1**	97.16	97.12	97.82	94.84	95.58	92.06	99.26	98.02	96.37	94.52	98.53	96.40	0.997
**2**	96.24	96.92	96.63	95.19	95.35	93.55	98.77	96.49	95.79	95.2	97.69	95.84	0.998
**3**	97.45	97.97	99.02	98.03	97.67	96.03	99.51	98.78	97.56	96.99	99.26	98.41	0.999
**4**	94.87	93.52	97.81	95.57	94.65	93.05	99.01	95.27	94.76	93.28	98.41	95.42	0.995
**5**	98.12	98.71	98.06	96.56	97.21	95.04	99.51	98.48	97.66	96.84	98.78	97.51	0.999
**6**	98.58	97.99	99.02	97.14	96.98	97.02	99.75	98.32	97.77	97.51	99.39	97.73	0.999
**7**	98.84	100.0	99.02	98.34	99.07	97.27	100.0	99.24	98.95	98.62	99.51	98.79	1.000
**8**	97.67	98.15	98.77	94.57	97.67	92.31	98.52	98.17	97.67	95.14	98.64	96.34	0.996
**Average (%)**	**97.35**	97.56	**98.22**	96.23	96.72	**94.51**	**99.29**	97.81	97.03	**96.01**	**98.75**	97.01	0.998
	**97.34**				97.08				97.2				

**Table 11:** Precision, recall, f1-score, and AUC for GRU model with 128 parameters.

Folds	Precision				Recall				f1-score				AUC
	ANG	EXC	SAD	NEU	ANG	EXC	SAD	NEU	ANG	EXC	SAD	NEU	
**0**	94.78	93.47	98.53	93.98	97.21	88.83	98.77	95.12	95.98	91.09	98.65	94.55	0.994
**1**	95.09	91.84	98.78	92.47	94.65	86.60	99.75	95.43	94.87	89.14	99.26	93.92	0.993
**2**	97.20	94.18	98.54	95.31	96.98	92.31	99.51	96.04	97.09	93.23	99.02	95.67	0.996
**3**	95.40	94.16	98.28	93.93	96.51	88.09	98.77	96.65	95.95	91.03	98.53	95.27	0.991
**4**	94.97	94.29	99.26	94.89	96.51	90.07	99.51	96.34	95.73	92.13	99.38	95.61	0.994
**5**	95.18	94.01	98.53	94.31	96.51	89.58	98.77	96.04	95.84	91.74	98.65	95.17	0.995
**6**	96.08	95.31	99.26	95.53	96.98	90.82	99.26	97.71	96.53	93.01	99.26	96.61	0.996
**7**	94.12	92.73	99.75	95.40	96.74	91.81	98.77	94.82	95.41	92.27	99.26	95.11	0.994
8	96.54	94.78	98.54	94.90	97.44	90.07	99.75	96.49	96.99	92.37	99.14	95.69	0.992
**Average (%)**	95.48	**93.86**	**98.83**	94.52	96.61	**89.80**	**99.21**	96.07	96.04	**91.78**	**99.02**	95.29	0.994
	**95.67**				95.42				95.53				

**Table 12:** Recapitulation of the average values of accuracy, precision, recall, f1-score, and AUC for 3 models with 128 (S1 set) and 153 (S2 set) feature parameters.

	Accuracy		Precision		Recall		f1-score		AUC	
	Highest	Lowest	Highest	Lowest	Highest	Lowest	Highest	Lowest	Highest	Lowest
S1 Model	97.18 **CRNN**	95.53 *GRU*	97.34 **CRNN**	95.67 *GRU*	97.08 **CRNN**	95.42 *GRU*	97.2 **CRNN**	95.53 *GRU*	0.998 **CRNN**	0.994 *GRU*
S2 Model	97.47 **GRU**	96.96 CNN	97.54 **GRU**	96.92 *CRNN*	97.38 **GRU**	96.64 *CRNN*	97.45 **GRU**	96.77 *CRNN*	0.9973 models	

**Table 13:** Accuracy, AUC and precision, recall, f1-score for each emotion for GRU model using 153 parameters without data augmentation.

Folds	Accuracy	Precision				Recall				f1-score				AUC
		ANG	EXC	SAD	NEU	ANG	EXC	SAD	NEU	ANG	EXC	SAD	NEU	
**0**	73.68	70.87	63.51	78.29	76.33	67.59	46.53	99.02	78.66	69.19	53.71	87.45	77.48	0.919
**1**	76.84	80.21	70.15	92.31	69.71	71.3	46.53	94.12	88.41	75.49	55.95	93.2	77.96	0.926
**2**	76.21	78	62.2	92.23	72.63	72.22	50.5	93.14	84.15	75	55.74	92.68	77.97	0.916
**3**	76.42	75.93	61.36	91.43	75.29	75.93	53.47	94.12	79.88	75.93	57.14	92.75	77.51	0.918
**4**	75.79	78.43	72.92	92.31	67.42	74.07	34.65	94.12	90.85	76.19	46.98	93.2	77.4	0.921
**5**	76.21	83.33	60.71	91.59	71.13	69.44	50.5	96.08	84.15	75.76	55.14	93.78	77.09	0.936
**6**	77.89	78.5	62.07	96.04	75	77.78	53.47	95.1	82.32	78.14	57.45	95.57	78.49	0.927
**7**	75.79	80.65	59.3	91.51	72.11	69.44	50.5	95.1	83.54	74.63	54.55	93.27	77.4	0.923
**8**	73.68	69.17	58.46	87.27	73.89	76.85	37.62	94.12	81.1	72.81	45.78	90.57	77.33	0.913
**Average (%)**	**75.83**	**77.23**	63.41	**90.33**	72.61	72.74	**47.09**	**94.99**	83.67	74.79	**53.6**	**92.5**	77.63	0.922
		75.9				74.62				74.63				

**Table 14:** Summary of research results on speech emotion recognition with IEMOCAP and for four emotions.

Ref.	Year	Model	Parameters	Average Accuracy (%)
[21]	2021	Acoustic Segment Model (ASM), DNN	Latent Semantic Analysis (LSA) with HMM for ASM	73.90
[27]	2021	PATHOSnet (Parallel, Audio-Textual, Hybrid Organisation for emotionS network)	Linguistic features + spectrogram	80.40
[29]	2021	SSA-CRNN-r (Self Speaker Attentive Convolutional Neural Network-regularization)	3-D Log-Mel spectrograms (with delta and delta-deltas)	95.90
[30]	2021	FaceNet	Spectrogram	68.96
[25]	2020	CRNN deep learning model based on Focal Loss	Spectrogram	69.33
[31]	2020	Deep stride convolutional neural network (DSCNN)	Spectrogram	81.75
[28]	2020	Combination of DCNN with a SincNet layer, RNN	Combined acoustic and textual data	80.51
[10]	2020	Hybrid architecture: DenseBlock + LSTM	Spectrogram	64.10
[32]	2020	DCNN	Spectrogram	83.80
[26]	2020	Attention-based Convolutional Neural Networks (ACNN)	MFCC	76.18
[11]	2020	LSTM	Log-spectra of short-time Fourier transforms (STFTs)	58.80
[22]	2020	CNN	Spectrograms and MFCC (Mel-Frequency Spectral Coefficients)	74.30
[17]	2020	Meta Multi-task Learning (MMTL), Meta Learner (CNN+LSTM) + Transfer Learner (Fully Connected Layer)	Spectrogram	76.64
[12]	2020	LSTM	MelSpectrogram	73.00
[13]	2020	1D convolutions + Bi-LSTM	Both audio and text information	72.82
[18]	2020	CNN, LSTM	3D log-Mel spectrograms	80.80
[23]	2020	DCNN	MFCC, chromagram, Mel-scale spectrogram, tonnetz, spectral contrast	64.30
[14]	2020	Bidirectional LSTM	Spectrogram	71.70
[35]	2019	Interaction-Aware Attention Network (IAAN)	MFCC, pitch	66.30
[15]	2019	DFF-ATMF (Deep Feature Fusion-Audio and Text Modality Fusion), LSTM	MFCC, spectral centroid, chroma stft, spectral contrast (Audio modality + text modality)	81.37
[24]	2019	CNN	Text & MFCC	76.10
[36]	2019	Emoception Network drawinginspiration from Inception Network	MFSC (Mel-Frequency Spectral Coefficients)	75.90
[16]	2019	Multi-head Self-attention+ Global Context-aware Attention Long Short-Term Memory recurrent neutral network (GCA-LSTM)	MFCC, F0, energy	79.20
[19]	2018	LSTM, CNN	MFCC, zero-crossing rate, short-term energy, short-term entropy of energy, spectral centroid and spread, spectral entropy, spectral flux, spectral rolloff	62.72
[33]	2018	Multi-channel CNN	Phoneme & Spectrogram	73.90
[34]	2018	Fully convolutional network (FCN) + Attention layer	Spectrogram	70.40
[20]	2017	CNN, Combined CNN, and LSTM	Spectrogram	68.00
[9]	2014	SVM	Low-level acoustic features and derivation, cepstral-based features, GMM supervectors	71.90
Our Research	2021	GRU, CRNN, CNN	153 parameters	**97.47**

## Data Availability

Not applicable.
